# Entry of Migrant Workers to Malaysia: Consideration to Implement Mass Drug Administration Against Intestinal Parasitic Infections

**DOI:** 10.34172/ijhpm.2024.7842

**Published:** 2024-04-24

**Authors:** Norhidayu Sahimin, Nabila Abu Bakar, Yvonne Ai Lian Lim, Jerzy Marian Behnke, John Lewis, Nurliana Kamaruddin, Siti Nursheena Mohd Zain

**Affiliations:** ^1^Tropical Infectious Diseases Research and Education Centre (TIDREC), Universiti Malaya, Kuala Lumpur, Malaysia.; ^2^Department of Parasitology, Faculty of Medicine, Universiti Malaya, Kuala Lumpur, Malaysia.; ^3^Institute of Biological Sciences, Faculty of Science, Universiti Malaya, Kuala Lumpur, Malaysia.; ^4^School of Life Sciences, University of Nottingham, University Park, Nottingham, UK.; ^5^School of Biological Sciences, Royal Holloway, University of London, Egham, UK.; ^6^Institute of Asia-Europe, Universiti Malaya, Kuala Lumpur, Malaysia.

**Keywords:** Urbanisation, Malaysia, Parasitic Infections, Anthelmintic Drugs, Migrant Workers

## Abstract

Over the last five decades, widespread industrialisation and urbanisation have resulted in the influx of low-skilled workers, particularly from Southeast and West Asia to Malaysia. The current practice for migrant workers entry for employment requires mandatory medical screening for infectious diseases. However, screening for parasitic infections in Malaysia is woefully inadequate. Many migrants come from low-income countries where parasitic infections are common, which may have public health implications for their overall well-being as parasitic infections, although not critical, may impact their overall productivity. The high prevalence of intestinal parasitic infections (IPIs) recorded among migrant workers in Malaysia necessitates improvement in the national health policy to include mandatory mass administration of a single dose of anthelmintic drugs to all low-skilled migrant labourers, particularly upon entry into the country, admission, and encourage continuous surveillance. A constant stream of migrant labourers is anticipated, potentially resulting in an ongoing occurrence of parasitic infections within the population. The implementation of economic measures like health awareness initiatives, routine deworming campaigns, and improved sanitation facilities holds the potential to reduce the spread of these infections notably. More often than not, taking preventive actions proves to be more financially efficient over time compared to addressing severe infections at a later stage.

## Background

**Key points**

- Intestinal parasitic infections (IPIs) are often overlooked; however, they can have severe consequences for infected subjects, including anaemia and bowel obstruction.
- The first report of non-endemic intestinal hookworm (*Ancylostoma duodenale*) comes from migrant workers in Malaysia.
- There is an urgency to implement and sustain regular deworming among the migrant workers when they first enter Malaysia and throughout their stay here.
- Deworming treatment with albendazole is cheap, effective, and deemed sustainable for long-term treatment.

According to the International Labour Organisation, 2019, there were roughly 169 million international migrant workers worldwide. Globalisation, demographic shifts, conflicts, financial inequities, and climate change are just a few of the factors that lead workers and their families to travel borders in search of better jobs and security.^1^ Malaysia has developed into a multi-sector economy in the late 20th and early 21st century, transitioning into a high-middle-income country. This resulted in the influx of low-skill workers annually to fill Malaysia’s low-skilled labour market, primarily in construction, domestic, food services, manufacturing, and plantation work.^2,3^ Many come from neighbouring countries with poor economic conditions and high poverty and unemployment rates, such as Indonesia, Bangladesh, Thailand, the Philippines, Pakistan, Myanmar, Nepal, India, Cambodia, Vietnam, Laos, and Sri Lanka.

###  The Push Factor

 Prospecting a better standard of life is a primary reason driving this migration trend to Malaysia. Up to 76% of the overall population has been urbanised, with an annual rate of change of 2.19% (2015–2020 established).^4^ In urban and rural regions, 96% of the population has access to basic sanitation facilities, and 98.2% has access to safe drinking water. Malaysia has a poverty rate of 5.6% (2018 established), significantly lower than most neighbouring countries. For example, Myanmar has the highest percentage of its population living in poverty (25.6%; 2016 established), followed by Nepal (25.2%; 2011 est.), Bangladesh (24.3%; 2016 est.), India (21.9%; 2011 est.), Indonesia (9.4%; 2019 est.) and Vietnam (6.7%; 2018 est.).^4^ Poor compensation and a lack of job prospects are the primary causes behind migration to Malaysia, where there are many job opportunities, appealing vacancies, and higher wages.^5^

###  Current Status of Migrant Workers in Malaysia

 Since the early part of the 21st century, the number of migrant workers has increased dramatically, from 1.06 million in 2002 to 1.8 million in 2017, with an all-time high of 2.1 million in 2015.^3^ Indonesians comprised up to 40.1% of the workers, followed by Nepalese (21.6%) and Bangladeshis (15.1%). All three nationalities worked mostly in manufacturing (35.9%), agricultural and plantation (23.4%), construction (19.8%), and domestic services (7.1%).^3^ Each foreign worker is subjected to a compulsory medical screening before and upon arrival in Malaysia. This is repeated annually until the third year of service under the same employer. Unitab Medic Sdn. Bhd. manages and supervises this obligatory screening programme on behalf of Foreign Worker’s Medical Examination Agency (FOMEMA). Furthermore, the programme assures that each worker’s health status includes being free from communicable diseases and having good physical fitness. This approach also protects Malaysia’s public health facilities, as extended medical care can potentially overload the heavily subsidised health system.

###  Potential Risks of Disease Outbreaks

 The Ministry of Health Malaysia mandated that the health screening process is carried out by a group of healthcare professionals in public health, occupational health, radiography, and laboratory services and includes the testing of blood and urine samples. The health screening covers many conditions, including the physical and medical histories of infectious diseases (Table). All registrations and payments are consolidated to facilitate the issuing of work permits, with medical reports, X-rays, and laboratory analyses provided separately and electronically to FOMEMA and the Malaysian Immigration Department. The results are obtained online, and only “fit” workers who have passed all the tests successfully can continue working in Malaysia.^6^

**Table:** List of Compulsory Medical Examination Categories for Workers Prior to Entry for Employment as Stipulated by the Ministry of Health

**Category**	**Examination **
Medical history	HIV/AIDS, tuberculosis, leprosy, viral hepatitis, peptic ulcer, epilepsy, Cancer, malaria, diabetes mellitus, hypertension, heart diseases, psychiatric illnesses, STDs, kidney disease, bronchial asthma, and others
Physical examination	Height and weight, pulse rate and blood pressure, last menstrual period (female), chronic skin rash, anaesthetic skin patch, anaemia, limb deformity, jaundice, lymph nodes enlargement, vision test, hearing ability, and others
System examination	Cardiovascular system, respiratory system, gastrointestinal system, nervous system, mental status, and genitourinary system
Laboratory tests	Blood test: For blood grouping (A, B, AB, or O and Rh) For HIV, hepatitis B, VDRL, and Malaria
	Urine tests: For colour, specific gravity, sugar, albumin, and microscopic examination For opiates, cannabis, and pregnancy (for female)
Chest X-ray	Physical examination of the foreign worker must be carried out first before chest X-ray examination

Abbreviations: STDs, sexually transmitted diseases; Rh, rhesus; VRDL, venereal disease research laboratory. Source: FOMEMA.^6^

 Yet, despite the possibility of parasite transmissions from recently arrived migrant workers from areas with inadequate hygiene and sanitation, screening for intestinal parasitic infections (IPIs) is currently not mandatory, thus posing health risks to the local population and the broader public. Therefore, the current study was carried out to highlight the importance of Mass Drug Administration to migrant workers prior to and upon admission into Malaysia based on previous analyses conducted amongst migrant workers in Malaysia.

###  Assessment of Intestinal Parasites Among Migrant Workers

 Data from several published and unpublished articles on migrant workers were assessed to highlight existing gaps in the health screening process and to suggest refinement to the current health policies, as shown in Figure.

**Figure:**
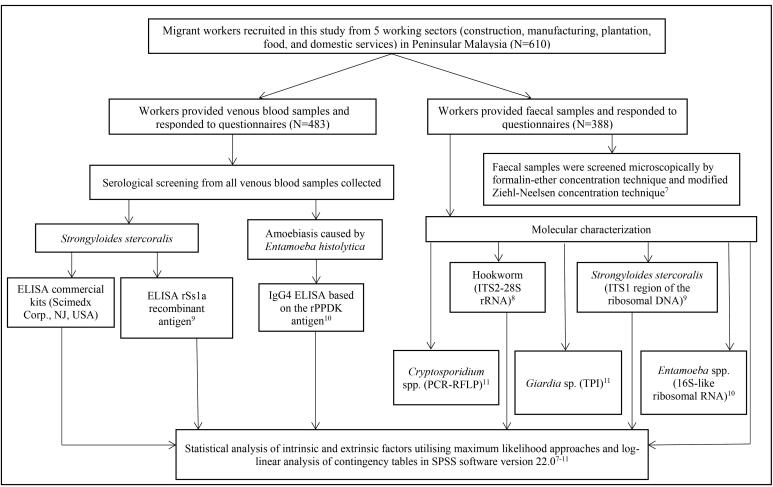
Recruitment, Sample Collection and Screening of Migrant Workers From 5 Working Sectors in Peninsular Malaysia.^7-11^ Abbreviations: PCR-RFLP, polymerase chain reaction-restriction fragment length polymorphism; TPI, triosephosphate isomerase; ITS1, internal transcribed spacer 1; rPPDK, recombinant pyruvate phosphate dikinase; IgG4, Immunoglobulin G4; ELISA, enzyme-linked immunosorbent assay.

 A study among low and semi-skilled employees showed a high prevalence of intestinal helminths such as *Ascaris lumbricoides *(43.3%) and hookworms (13.1%), with lower levels of *Trichuris trichiura *(9.5%), *Enterobius vermicularis *(0.5%) and *Hymenolepis nana *(1.8%).^7^ Overall, two risk factors, nationality and years of residency in the country, influenced infection levels, with higher prevalence occurring in Nepalese and newly arrived workers.^7^ The presence of two species of hookworms (*Necator americanus *and *Ancylostoma duodenale) *was recorded.^8^ It is important to note that this is the first record of *A. duodenale *in Malaysia, coming from newly arrived Nepalese and Indonesian workers. On the other hand, another study^9^ on the epidemiology of strongyloidiasis amongst migrant workers showed varying prevalence depending on the techniques used: stool examination (no detection), commercial Immunoglobulin G–enzyme-linked immunosorbent assay (IgG-ELISA) (35.8%), rSs1a-ELISA (13.0%) and nested polymerase chain reaction (0.8%), which highlights the importance of using appropriate diagnostic tools for detection. To date, studies on strongyloidiasis among Malaysian populations have been of minor concern and limited to the Orang Asli (aborigines) communities.^12-15^

 In addition, three parasitic protozoan species were recovered from stool samples, namely *Entamoeba *spp. (11.6%), *Giardia *spp. (10.8%) and *Cryptosporidium *spp. (3.1%).^7^ Molecular amplification of the gene also resulted in the identification of two *Entamoeba *species; *Entamoeba dispar *(5.9%)and* Entamoeba histolytica *(2.8%), with mixed infections reported in three samples.

###  Issues with Soil-Transmitted Helminths and Neglected Tropical Diseases

 Soil-transmitted helminths (STHs) are collectively identified as one of 17 neglected tropical diseases worldwide. The term ‘neglected’ describes various factors, including the limited attention given to these diseases by policymakers, a lack of priority within health strategies, inadequate research, limited resource allocations and few interventions.^16^ Up to 24% of the world’s population, comprising 1.5 billion people, are infected with intestinal nematode species such as the roundworm, whipworm, and hookworms, where transmission occurs primarily through soil contaminated with human faeces.

 Neglected IPIs such as STH have been recognised as one of the main causes of illnesses, especially among vulnerable communities.^17,18^ Transmission of parasitic infections predominantly depends on human behaviour, particularly during ingestion, defecation, and subsequent levels of personal hygiene. In varying degrees, these infections can cause anaemia, vitamin A deficiency, stunted growth, malnutrition, intestinal obstruction, and impaired development in children, including delayed maturation of cognitive skills.^19^ Globally, up to 800 million people are known to harbour roundworms,^17^ compared to 600 million people with whipworm and hookworm infections.^20,21^

###  Human Intestinal Parasitic Infections in Malaysia

 Parasitic infections continue to be a public health issue, especially amongst poverty-stricken communities^18^ with varying prevalences amongst the Orang Asli (44.3-99.2%),^17,18,22-26^ plantation and rural communities (32.3%-70.0%),^27-30^ slum dwellers (20.6%-90.9%),^18,31^ fishing communities (54.2%-98.0%),^32-36^ and flat dwellers (5.1%-57.0%).^8,37-39^

 Nevertheless, the introduction of mass drug administration in the 1970s and improvements in living condition has resulted in a dramatic decline in infection levels amongst slum dwellers (90.9% in 1978 to 20.6% in 2014),^18,31^ flat dwellers (57% in 1983 to 5.5% in 2014),^18,37^ and rural communities (90.0% in 1970 to 32.3% in 2014).^18,27^

 However, despite providing basic accommodation and facilities that include clean water and flush toilets to migrant workers in the country, the infection levels of *A. lumbricoides, T. trichiura* and *N. americanus* continue to persist. These infections are evident particularly among newly arriving workers within a year of residency. It is clear that these infections are exacerbated through human behaviour, particularly during ingestion and defecation.

###  Policy Recommendation and Working Strategy

 The present findings suggest that the health screening process and current health policies in the case of migrant workers require some refinement and that a mass drug administration using a single dose of albendazole (400 mg) be recommended for newly arrived workers upon entry to Malaysia to control IPIs as stated by the World Health Organization (WHO) in 2001.^16^ In Malaysia, the occurrence of high prevalence of parasitic infections amongst previous cohorts of migrant workers^7,8,11^ provides an insight into the poor conditions under which they live, often linked with unacceptable hygiene practices and inadequate sanitation.

 Albendazole is inexpensive, easy to administer by non-medical personnel when entering Malaysia, and particularly effective against intestinal nematode and protozoan infections.^16,40,41^ As a blanket rule, the WHO recommends periodic treatment (de-worming) without a previous individual diagnosis to all at-risk people living in endemic areas. Treatment should be given once a year when the baseline prevalence of STH infections in the community is above 20% and twice a year when the prevalence of STH infections in the community is above 50%.^42^

 Such a requirement is already implemented in countries dependent on immigrant workforces. For example, in Qatar, prospective workers must undergo health checks at approved clinics in their country of origin, and if infections are detected, albendazole is administered prior to arrival as a condition of entry and the issuance of a work permit.^43,44^ In addition, those working in the food service industry need to undergo annual compulsory examinations organised by the Medical Commission as a condition for continuation of their work permits. It was also reported that infections among newly arrived migrant workers were higher than those who had previously stayed and worked in the city.^45^ The Thai government also implemented the same requirement to screen their workers before emigration for IPIs, and an entry permit will not be issued for those who failed the requirement.^46^

 Therefore, the provision of mass drug administration will not only benefit individual workers by ensuring a better quality of life, but the likelihood of higher productivity in the workforce will positively impact the government, prospective employers, and the general population.

 Hence, sustained monitoring and the introduction of mandatory chemotherapy using metronidazole may be warranted in the future if infection levels of the protozoan species increase. For entry to Malaysia, requirements should focus on regularly monitoring migrant workers for STHs and providing anthelmintic treatment where necessary. In addition, environmental and public health programs should be introduced to show why knowledge and understanding of disease transmission are fundamentally linked with satisfactory standards of personal hygiene and sanitation.

## Conclusion

 The continuing high prevalence of IPIs amongst migrant workers calls for refining the present health policy for migrant workers in Malaysia. This includes mandatory mass drug administration in newly arrived workers, continued monitoring of intestinal protozoan infections to safeguard against potential threats from infective species, and metronidazole for the latter as circumstances demand. These measures should be accompanied by the introduction of health campaigns or programs to increase community awareness and understanding of how improved levels of personal hygiene, sanitation, cleanliness, and healthy behaviour can lead to the prevention and control of parasitic infections.

## Acknowledgements

 The authors wish to extend their grateful thanks to the Ministry of Health, Malaysia and all collaborators from companies and agencies for their cooperation and support in this study. Special thanks are also extended to all medical staff and nurses from the University Malaya Medical Centre and Hospital Universiti Kebangsaan Malaysia (HUKM) for their technical assistance. Most importantly, the authors would like to thank all participants who voluntarily took part in this study.

## Ethical issues

 This study obtained the ethical clearance of the University of Malaya Medical Centre (UMMC), Malaysia (reference number: MECID NO: 20143-40).

## Competing interests

 Authors declare that they have no competing interests.

## Funding

 This work was funded by grants from the Ministry of Higher Education under the Fundamental Research Grant Scheme (FRGS) (FRGS/1/2020/SKK0/UM/02/18); Swiss Tropical and Public Health Institute (IF004-2021); Fundamental Research Grant Scheme (FRGS) from Ministry of Higher Education, FP015-2014B; and UM/MoHE High Impact Research Grant (UM.C/625/1/HIR/MOHE/MED/23).
